# PPAR-γ Activation Alleviates Intestinal Dysfunction and Lactose Malabsorption in Experimental Food Allergy Rats

**DOI:** 10.3390/nu18040653

**Published:** 2026-02-16

**Authors:** Yuyang Hao, Lu Yao, Yuxin Jin, Sheng Yin, Zhiwei He, Huilian Che

**Affiliations:** 1 College of Food Science and Nutritional Engineering, China Agricultural University, Beijing 100080, China; yyhao@cau.edu.cn (Y.H.); j17860722880@163.com (Y.J.); 2 Key Laboratory of Food Nutrition and Health Evaluation Technology, State Administration for Market Regulation, Beijing 100080, China; 3 School of Microbiology, University College Cork, T12 K8AF Cork, Ireland; 4 School of Food & Health, Beijing Technology & Business University, Beijing 100048, China; yinsheng@btbu.edu.cn; 5 School of Science, China Agricultural University, Beijing 100080, China; hezw@cau.edu.cn

**Keywords:** PPAR-γ, food allergy, lactose, SGLT1, GLUT2

## Abstract

Background/Objectives: Food allergy-induced intestinal inflammation can impair lactose digestion and absorption by damaging the epithelium, leading to secondary lactase deficiency with no effective treatments. The immunometabolism nuclear receptor PPAR-γ regulates gut epithelial function and nutrient absorption. This study aimed to determine whether PPAR-γ activation can preserve lactose digestion and absorption during allergic inflammation and to elucidate the underlying mechanisms. Methods: In an ovalbumin-sensitized Brown Norway rat model of food allergy, animals were treated with either the PPAR-γ agonist rosiglitazone or the antagonist GW9662. Lactose absorption was assessed by in vivo lactose tolerance tests (blood glucose monitoring) and intestinal transit measurements. Jejunal tissues were analyzed for lactase gene expression, lactase enzyme activity, and SGLT1/GLUT2 transporter levels. Results: Allergic rats exhibited reduced weight gain, delayed intestinal transit, and lactose malabsorption (lower blood glucose after lactose challenge), accompanied by sharply decreased jejunal lactase mRNA, enzyme activity, and SGLT1/GLUT2 levels. Rosiglitazone treatment restored intestinal PPAR-γ expression and markedly improved lactose absorption, normalizing the lactose tolerance curve. Rosiglitazone also increased lactase gene expression and enzyme activity, and upregulated SGLT1 levels. In contrast, PPAR-γ inhibition with GW9662 further reduced lactase and transporter levels and failed to improve absorption. Conclusions: PPAR-γ signaling maintains intestinal lactose digestive capacity of rats during allergic inflammation by sustaining lactase production and monosaccharide transporter expression. Our findings verify an immunometabolism mechanism linking nuclear receptor activation to enhanced nutrient absorption and highlight PPAR-γ agonism as a promising therapeutic strategy to alleviate food allergy-associated lactose malabsorption.

## 1. Introduction

Food allergy (FA) is a growing global public health concern characterized by a breakdown of immunological tolerance to ingested food antigens [1,2]. The most severe manifestation of FA is anaphylaxis, an immediate multi-organ reaction that can induce life-threatening hypovolemic shock and respiratory compromise [3]. Epidemiological surveys indicate that FA primarily affects children and infants, with a prevalence of up to ~10% in developed countries [4,5,6,7]. FA can present with diverse symptoms and often involves the gastrointestinal tract. In some cases, particularly when FA causes chronic non–IgE-mediated gut inflammation, an allergic enteropathy develops that damages the intestinal mucosa and results in secondary lactase deficiency—a transient lactose malabsorption that is frequently overlooked despite its clinical significance [8,9]. Affected infants may experience chronic diarrhea, bloating, and malnutrition due to the inability to properly digest lactose [10]. Clinical reports note decreased growth in 0–3-year-old children with FA compared to healthy children [11]. Currently, no specific therapeutic guidelines exist for managing secondary lactase deficiency in FA beyond dietary elimination and gradual reintroduction, highlighting an unmet need for clear strategies in management.

Peroxisome proliferator-activated receptor gamma (PPAR-γ) is a member of the nuclear receptor family, a transcription factor which is central to lipid and glucose metabolism, adipogenesis, and immune regulation [12]. To date, genomic mapping stimulated by PPAR-γ agonists in intestinal epithelial cells (IECs) and functional studies in mice have begun to reveal the role of this receptor in the intestine [13]. Recent evidence suggests that enhancing PPAR-γ activity can attenuate intestinal allergic inflammation. In an ovalbumin-induced food allergy model, a PPAR-γ agonist (rosiglitazone) markedly reduced clinical symptoms and mucosal IgE levels, while preserving tight junction integrity. PPAR-γ activation shifted the immune milieu toward a less Th2-dominated response (lower IL-4/IL-5 and higher IFN-γ) and directly inhibited mast cell degranulation [14]. A few studies report that PPAR-γ influences nutrient digestion and absorption [15,16]. PPAR-γ agonists induce the expression and activity of the lactase (LCT) enzyme in enterocytes [17], and the loss of insulin-stimulated glucose transport into 3T3-L1 adipocytes is due to a decrease in GLUT1 and GLUT4 function caused by PPAR-γ decrease [18]. However, under allergic inflammatory conditions, PPAR-γ signaling is often suppressed, yet there is an unmet need to clarify whether PPAR-γ’s beneficial roles in lactose metabolic disorders extend to conditions of allergic inflammation.

Against this background, we established a BN rat model of food allergy to analyze lactose digestion and absorption under allergic conditions and the relationship between the PPAR-γ signaling pathway and this process. Additionally, we examined how PPAR-γ regulates the activity and expression of rat intestinal LCT and sodium-dependent glucose transporters (GLUT1 and SGLT2) at the gene and protein levels, aiming to identify the connection between PPAR-γ signaling and lactose metabolic disorders potentially accompanying food allergies. Our findings confirmed that the activation of PPAR-γ signaling could partially restore intestinal function and lactose metabolism impaired by food allergies. Ultimately, this work could identify PPAR-γ as a novel therapeutic target for preserving gut function in food allergy while dampening pathological immune responses.

## 2. Materials and Methods

### 2.1. The Experimental Animals

Four-week-old SPF-grade female Brown Norway (BN) rats were purchased from Charles River Laboratories Inc., Beijing, China (License Number: SCXK(Jing)-2016-006), and housed in an SPF-grade animal facility (License Number: SYXK(Jing) 2020-0052). All rats were female; the estrous cycle phase was not controlled during the study. Throughout the study, the rats had ad libitum access to food and water. Environmental conditions were maintained at a temperature of 23 ± 3 °C, with humidity levels between 40% and 70%, and a 12 h light/dark cycle. All animal experiments were conducted under protocols approved by the Animal Welfare and Ethics Review Committee of China Agricultural University (Project Identification Code: AW30109102-4) and were in compliance with the ethical standards of the university. Efforts were made to minimize animal suffering, reduce the number of animals used, and employ in vivo alternative techniques where possible.

### 2.2. Protocol for Animal Experiment

**Figure 1:**
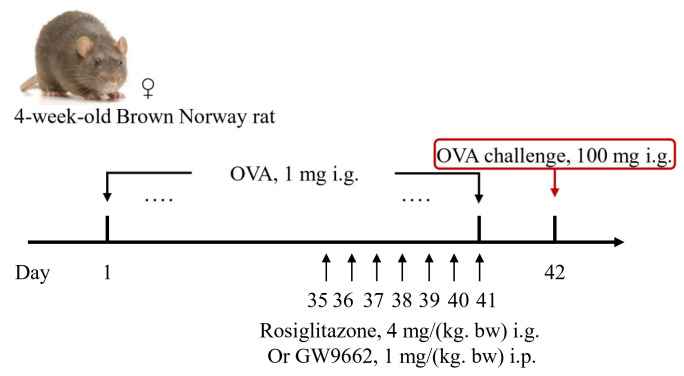
Flow Chart of Establishing Food-Allergic Rat Model. Note: i.p.: intraperitoneal injection; i.g.: oral gavage. The ellipsis (“.…”) indicates repeated OVA administration (1 mg, i.g.) on days 1–41.

After one week of acclimatization, a total 40 BN rats were randomly divided into four groups (*n* = 10 per group): negative control group, OVA-sensitized group, OVA-sensitized + PPAR-γ agonist (Rosi)-treated group, and OVA-sensitized + PPAR-γ inhibitor (GW9662)-treated group. Sample sizes were determined a priori based on ethical considerations and the effect sizes observed in preliminary experiments [14]. Between days 0 and 35, each rat was orally administered 600 μL of saline solution containing 1 mg OVA daily, without adjuvant. On day 42, a high-dose challenge was performed, where each rat received 100 mg OVA. From days 35 to 41, rats in the PPAR-γ agonist-/inhibitor-treated groups were orally administered Rosi or intraperitoneally injected with GW9662 daily (Figure 1). Rosi was administered via oral gavage at 4 mg/kg/day (dose based on [19]); GW9662 was administered via intraperitoneal injection at 1 mg/kg/day (dose based on [20]). Both drugs (Rosi and GW9662) were dissolved in a phosphate-buffered saline (PBS) containing 20% DMSO, which was used as a solubilizing co-solvent, as prior rodent studies indicate tolerability at comparable exposures without significant effects on intestinal motility [21]. Meanwhile, the negative control group received 600 μL of saline, and the OVA-sensitized group continued with 600 μL saline containing 1 mg OVA. The food allergy rat model used in this study was successfully established and validated, employing rats from the same experimental cohort as described previously [14]. Treatments and outcome measurements were performed in a random order across groups. All measurements were conducted at a consistent time of day by the same operator.

### 2.3. Lactose Digestion and Glucose Absorption Test in Rats

During the modeling period, body weight and food intake were measured. One day before the oral high-dose allergen challenge, three rats from each group were randomly selected to receive either 600 μL of lactose (40 mg/rat) or 600 μL of glucose (20 mg/rat). Blood samples were collected from the tail tip at intervals of 7.5, 15, 30, 60, 120, and 180 min post-administration to measure blood glucose levels.

### 2.4. Rat Intestinal Motility Experiment

Intestinal transit was measured 3 days after the high-dose challenge on day 42 to allow acute allergic reaction symptoms to abate, thereby assessing persistent changes in motility; three rats from each group were randomly chosen for an ink propulsion test. Animals in each group were orally administered a mixture of 10% activated charcoal and 5% gum arabic suspension (10 mL/kg). After 25 min, rats were euthanized by cervical dislocation, and the gastrointestinal tract was isolated and placed on a clean flat surface. The full length of the duodenum to the ileocecal junction and the length covered by the ink were measured. The ink propulsion percentage was calculated using Formula (1):

(1) $$\mathrm{Ink}\;\mathrm{Propulsion}\;\mathrm{Percentage}=\frac{\mathrm{Distance}\;\mathrm{from}\;\mathrm{the}\;\mathrm{front}\;\mathrm{of}\;\mathrm{the}\;\mathrm{ink}\;\mathrm{to}\;\mathrm{the}\;\mathrm{pylorus}}{\mathrm{total}\;\mathrm{length}\;\mathrm{of}\;\mathrm{the}\;\mathrm{small}\;\mathrm{intestine}}\times 100\%$$

### 2.5. Real-Time Quantitative Polymerase Chain Reaction (qRT-PCR)

All equipment and materials for RNA extraction (10 μL, 200 μL, 1 mL pipette tips, 100 μL and 1 mL centrifuge tubes, mortars, scissors, etc.) were treated for decontamination by immersion in 1% DEPC water overnight and subsequently dried at 150 °C for over 4 h. Before grinding tissues, the mortar was precooled with liquid nitrogen, and then the tissues were quickly transferred and ground to a powder-like consistency, constantly replenished with liquid nitrogen. For every 100 mg of tissue, 1 mL of TransZol Up was added, and grinding continued before transferring to a nuclease-free centrifuge tube. Next, 0.2 mL of chloroform was added, and the mixture was inverted several times for mixing. After letting it stand for 10 min at room temperature, it was centrifuged at 12,000× *g* at 4 °C for 15 min. The aqueous phase was transferred to a new nuclease-free tube, followed by the addition of an equal volume of isopropanol (0.5 mL). After inverting to mix and letting stand for 10 min at room temperature, it was centrifuged again under the same conditions. The RNA pellet was washed several times with 75% ethanol prepared in DEPC water and centrifuged at 12,000 rpm at 4 °C for 8 min. After allowing the pellet to air-dry, it was resuspended in 50–100 uL of DEPC-treated water to obtain the RNA. cDNA was synthesized using the Fast Quant cDNA First Strand Synthesis Kit (TIANGEN BIOTECH (BEIJING) CO.,LTD., Beijing, China) by adding 1500 ng of RNA. Quantitative RT-PCR was performed using the Quant qRT-PCR kit (SYBR Green) with a cycling program of 95 °C for 60 s, followed by 40 cycles of 95 °C for 5 s and 60 °C for 30 s. The relative mRNA expression levels of target genes in each sample were calculated using the following formula: relative mRNA expression = 2^-ΔCt^ × 100%, where ΔCt = target gene Ct—reference gene (β-actin) Ct. Primer sequences are provided in Table 1.

### 2.6. Lactase Activity Assay

Lactase activity was assessed using the glucose oxidase method previously described by Andrew et al. [22]. Jejunal tissue samples were homogenized on ice in 0.9% NaCl, then diluted in 0.9% NaCl (1/500), and 50 μL of the diluted solution was incubated with lactose buffer for 1 h. The supernatant was collected and incubated with 100 μL of glucose oxidase for 1 h. The reaction was terminated by adding 100 μL of H_2_SO_4_, and absorbance was measured at 450 nm. For each experiment, background interference was eliminated by incubating tissue cell extracts in lactose-free buffer.

### 2.7. Analysis of LCT, GLUT2, and SGLT1 Expression in Small Intestinal Tissue (Western Blot)

Protein extraction: (1) Approximately 50–70 mg of small intestine tissue was rapidly ground in a mortar with liquid nitrogen. (2) The lysed solution was then added to a volume of 200–500 mL of RIPA lysis buffer containing 1% protease inhibitor (PMSF), depending on tissue size. Samples were lysed on ice for at least 20 min, followed by cell disruption on ice for 3 times, 3 s each. (3) After centrifuging at 14,000× *g* at 4 °C for 15 min, the supernatant, which contains the proteins, was collected. Protein concentration was determined using the BCA method and adjusted according to experimental needs, and then stored at −20 °C. The Western blot method was based on protocols from previous publications of our group [14].

### 2.8. Immunofluorescence

Notably, 24 h after the day 42 high-dose OVA challenge, rats were humanely euthanized for sample collection (including jejunum tissues for immunofluorescence and other analyses). Jejunum tissues were excised and fixed in 4% PFA. After processing, including dehydration, embedding, and sectioning, immunofluorescence staining for LCT, GLUT2, and SGLT1 was performed. Tissue sections were then visualized under a fluorescence microscope.

### 2.9. Inclusion and Exclusion Criteria

Animals were included if they remained clinically healthy after acclimatization and completed the study protocol. Exclusion criteria were defined a priori and included procedural failure (e.g., unsuccessful gavage or injection) and severe distress requiring humane euthanasia. No adverse events, including unplanned mortality, occurred; therefore, no animals were excluded. All collected data were analyzed, and no data points were removed.

### 2.10. Integrated Optical Density (IOD) Analysis

Immunofluorescence images were acquired using identical exposure and gain settings across groups within each staining run. Quantification was performed in Fiji (version of ImageJ; NIH). For each animal, 3–5 non-overlapping jejunal fields were analyzed. Regions of interest (ROIs) were drawn over the villus epithelium, and the background was measured in an adjacent area and subtracted. IOD was calculated as Formula (2):

(2) IOD = (mean gray value background) × ROI area

The mean IOD per animal was used for statistical analysis.

### 2.11. Data Analysis

All data are presented as mean ± SEM from three independent biological replicates. Statistical differences were assessed by one-way ANOVA, followed by LSD post hoc tests in GraphPad Prism 10.1.2 (GraphPad Software, Boston, MA, USA), with *p* < 0.05 considered significant.

## 3. Results

### 3.1. Restorative Effects of PPAR-γ Signaling Activation on Food Intake, Body Weight, and Intestinal Motility of Food-Allergic Rats

**Figure 2:**
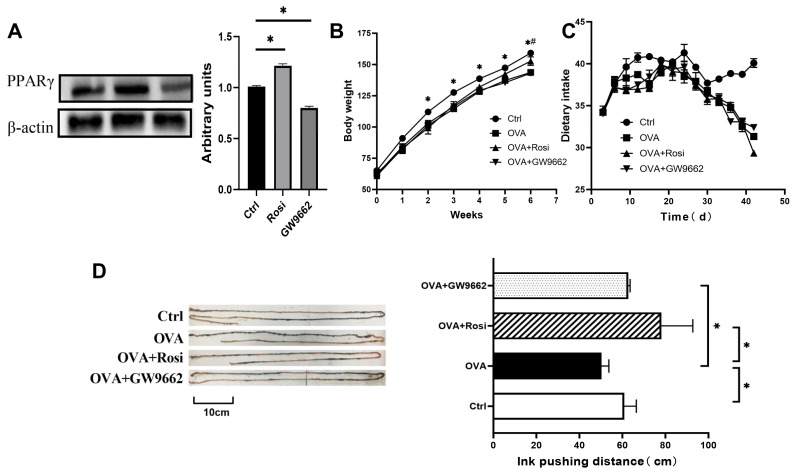
Restorative effects of PPAR-γ signaling activation on intestinal barrier damage and nutrient absorption in food allergic rats. (**A**) Expression of PPAR-γ in rat intestine after Rosi and GW9662 treatments. (**B**) Effect of PPAR-γ signaling on body weight in rats. (**C**) Effect of PPAR-γ signaling on food intake in rats. (**D**) Changes in intestinal motility in rats. Note: Ctrl: control group; Rosi: PPAR-γ agonist Rosi treatment group (Rosi treated for one week); GW9662: PPAR-γ inhibitor GW9662-treated group (GW9662 treated for one week); Figure * denotes *p* < 0.05 OVA-sensitized rats versus Ctrl group, and # denotes *p* < 0.05 for OVA-sensitized group compared with Rosi group; Data are presented as mean ± SEM (*n* = 3).

To assess the role of PPAR-γ signaling in food-allergic rats, we administered the PPAR-γ agonist Rosi and the PPAR-γ antagonist GW9662 to BN rats for one week. The expression of PPAR-γ in the rat intestine post-treatment is illustrated in Figure 2A. Rosi effectively upregulated the expression of PPAR-γ in the rat jejunum, while GW9662 exerted an opposite effect.

Then, we established a food-allergic rat model via OVA gavage. Rats were treated with either Rosi or GW9662. Throughout the study, food intake and body weight were monitored to preliminarily determine the effects of the PPAR-γ signaling pathway on rat nutrient absorption. The results revealed a retardation in weight gain in OVA-sensitized rats, andsignificant differences in body weight were observed between the OVA-sensitized and control groups from week 2 onwards (*p* < 0.05). By week 6, Rosi treatment raised the weight of sensitized rats to a level significantly higher than that of the untreated OVA group (*p* < 0.05), approximating the weight of the negative control rats prior to euthanization (Figure 2B). Previous studies from our group indicated that during food allergies, the intestinal barrier of rats is compromised, and substantial damage was observed in the intestinal lamina propria [23,24]. However, Rosi treatment upregulates the expression of tight junction proteins and reduces the penetration of food antigens [14]. So, we postulate that the activation of PPAR-γ may enhance rat digestion and absorption by restoring the intestinal barrier function. In terms of food intake (Figure 2C), the three sensitized groups exhibited consistent data trends in both food intake and weight, displaying significant reductions in food intake due to food allergies. Moreover, while PPAR-γ activation slightly elevated rat food intake, and PPAR-γ inhibition exacerbated anorexia in rats (*p* > 0.05 for both), although neither change was statistically significant.

Gastrointestinal motility can reflect the metabolic status of nutrients within the organism [25]. Thus, we proceeded to assess intestinal motility. Compared to the control group, the intestinal ink propulsion distance percentage in the OVA-sensitized rats significantly decreased (*p* < 0.05) (Figure 2D), indicating marked impairment in their intestinal motility. To clarify, gastric emptying is an upstream determinant of oral charcoal/ink propulsion measurements. In sensitized rats, intraluminal antigen challenge has been reported to delay gastric emptying in association with mucosal mast cell activation and reduced antral contractility [26,27]. Since gastric emptying was not measured in the present study, we interpret the propulsion readout as reflecting the combined effects of upper gastrointestinal delivery and small-intestinal propulsion, and we note this as a limitation. However, according to the higher intestinal ink propulsion distance percentage in the OVA-Rosi group than in the OVA+GW9662 group (*p* < 0.05), there is no doubt that activation of the PPAR-γ signaling pathway significantly enhanced the intestinal ink propulsion distance in rats, suggesting its beneficial role in restoring intestinal motility, which may also favor nutrient metabolism.

### 3.2. The Modulation of PPAR-γ Signaling Impacts Lactose Metabolism in Food-Allergic Rats

**Figure 3:**
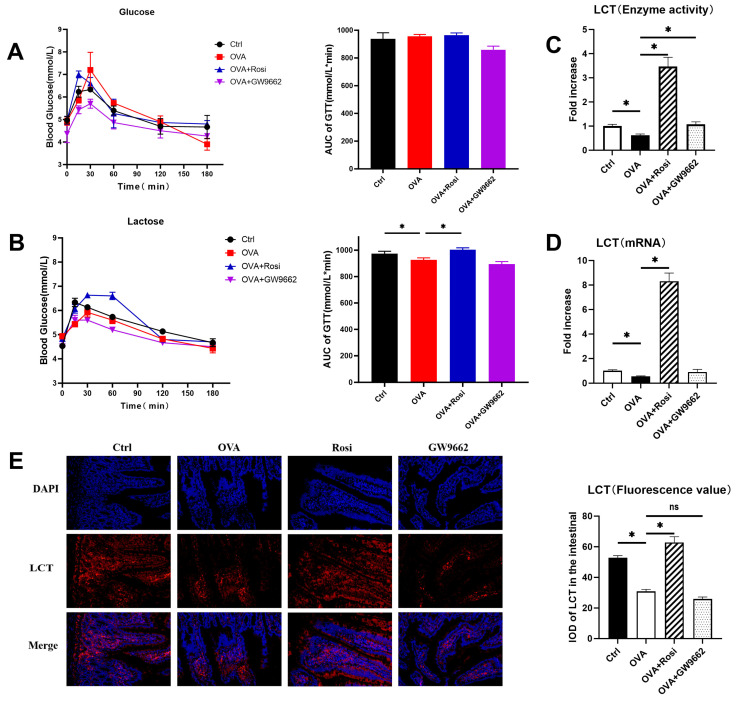
PPAR-γ modulates LCT and impacts lactose metabolism in OVA-sensitized allergic rats. (**A**) Changes in glucose tolerance in rats. (**B**) Changes in lactose tolerance in rats. (**C**) Enzyme activity of lactase in rat jejunum. (**D**) mRNA level of lactase in rat jejunum. (**E**) Fluorescent staining of expression and transcription levels of lactase in rat jejunum. Tissue slide magnification: 200×. Immunofluorescence signals were quantified by integrated optical density (IOD). Note: Ctrl: control group; OVA: OVA-sensitized group; OVA + Rosi: PPAR-γ agonist Rosi treatment group (Rosi treated for one week); OVA + GW9662: PPAR-γ inhibitor GW9662-treated group (GW9662 treated for one week); Nuclei were counterstained with DAPI (blue); LCT are shown in red; merged images are shown in the bottom row; Graph * indicates *p* < 0.05, ns indicates non-significant, *n* = 3.

To investigate the potential effects of PPAR-γ on lactose metabolism and glucose absorption, we employed glucose tolerance tests [28]. As there were no significant changes in the area under the curve (AUC) of each group (*p* > 0.05), neither allergy nor PPAR-γ modulation significantly affected glucose absorption in rats (Figure 3A). However, data on lactose tolerance presented a different trend. Compared to healthy rats, we observed a slightly decreased lactose metabolism curve in allergic rats, with a significant decline in its AUC (*p* < 0.05). Moreover, activation of PPAR-γ signaling notably ameliorated lactose metabolism (*p* < 0.05) (Figure 3B), suggesting a mechanism through which lactose absorption is enhanced during food allergy. In contrast, inhibition of PPAR-γ signaling by GW9662 did not have a significant effect on lactose metabolism. Collectively, these results imply that PPAR-γ signaling activation may alleviate lactose metabolic disturbances under food-allergic conditions. The precise regulatory mechanism warrants further exploration.

To further elucidate the mechanism underlying PPAR-γ-mediated lactose metabolism regulation, we measured the expression and activity of LCT, the most representative enzyme for lactose metabolism in the rat small intestine [29], in rat jejunum tissues using RT-qPCR, immunofluorescence staining, and enzyme activity assays (Figure 3C). OVA-induced allergic rats exhibited significantly reduced LCT mRNA expression, enzyme activity, and protein levels compared to the control group (*p* < 0.05). Treatment with the PPAR-γ agonist rosiglitazone (OVA + Rosi) significantly elevated LCT mRNA transcription (Figure 3D), enzyme activity, and protein expression in allergic rats compared with untreated allergic animals (*p* < 0.05) (Figure 3E). In contrast, treatment with GW9662 (OVA + GW9662) led to a significant suppression of LCT expression in the jejunum (*p* < 0.05), and correspondingly, failed to increase LCT mRNA and enzyme activity levels significantly compared to Rosi treatment. Immunofluorescence analysis confirmed that allergic inflammation markedly reduced LCT protein expression, whereas Rosi effectively restored its expression levels. Together, these data demonstrate that the lactose metabolism via LCT was destroyed in food allergy, while it was reversed by activating PPAR-γ signaling.

### 3.3. PPAR-γ Signaling Modulates Glucose and Galactose Transporter Expression in Food-Allergic Rats

**Figure 4:**
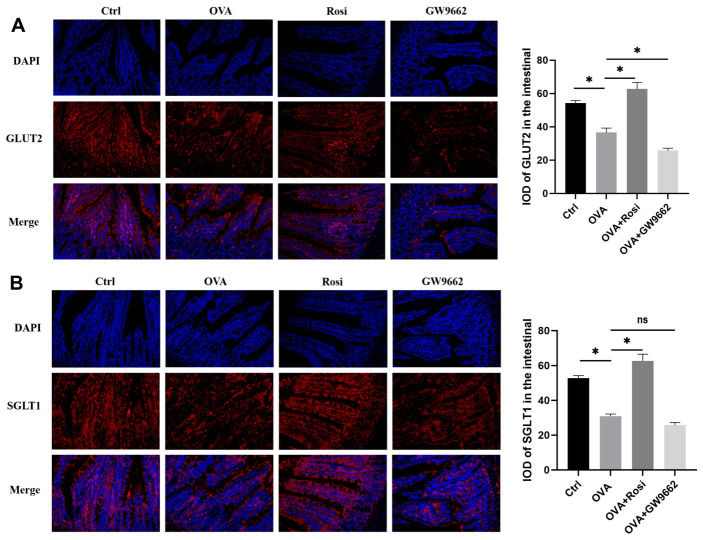
PPAR-γ signaling modulates glucose and galactose transporter expression in food allergic rats. (**A**) Expression of GLUT2 in rat jejunum. (**B**) Expression of SGLT1 in rat jejunum. Note: Ctrl: control group; OVA: OVA-sensitized group; OVA + Rosi: PPAR-γ agonist Rosi treatment group (Rosi treated for one week); OVA + GW9662: PPAR-γ inhibitor GW9662-treated group (GW9662 treated for one week); Nuclei were counterstained with DAPI (blue); GLUT2/SGLT1 are shown in red; merged images are shown in the bottom row; Graph * indicates *p* < 0.05, ns indicates non-significant, *n* = 3. Tissue slide magnification: 200×; immunofluorescence signals were quantified by integrated optical density (IOD).

We investigate the transport of lactose hydrolysis products. The monosaccharides galactose and glucose generated from lactose hydrolysis require active transport via specific receptors for absorption by the body. Key players in this transport process are the glucose transporter type 2 (GLUT2) and the sodium-dependent glucose co-transporter (SGLT1) [30,31]. In Figure 4A,B, we observed that the expression levels of both GLUT2 and SGLT1 in allergic rats were significantly decreased compared to the control group (*p* < 0.05). Activation of PPAR-γ signaling prominently upregulated the expression levels of SGLT1 (*p* < 0.05), while only modestly enhancing GLUT2 expression. Treatment with GW9662 displayed opposite effects. These results indicate that PPAR-γ signaling activation primarily boosts SGLT1 levels, facilitating the transport of glucose and galactose under food-allergic conditions.

## 4. Discussion

Prior studies from our research group identified histological features in the intestines of food allergic subjects, such as villous atrophy, mucosal erosion, and infiltration by inflammatory cells [32]. These histological alterations suggest a loss of intestinal epithelial function, yet the absorptive capacity of IECs under conditions of food allergy remains unclear. Numerous studies suggest that mucosal inflammation correlates with the expression of enzymes and transport proteins involved in nutrient absorption [33,34], indicating potential malabsorption issues due to intestinal inflammation. In this study, we observed that rats with food allergies experienced markedly reduced food intake and weight gain compared to control groups since week 2. This pattern likely stems from the compounded effects of inflammatory responses and epithelial dysfunction, highlighting the immediate nutritional challenges faced by patients with FA. In contrast, allergic rats treated with a PPAR agonist showed a progressive recovery in body weight and approached the level of negative controls by week 6, suggesting that activation of PPAR signaling may exert beneficial effects on nutrient absorption. Gastrointestinal motility provides a more sensitive readout of overall nutritional and metabolic status; activation of PPAR-γ signaling significantly increased the intestinal transit distance in the ink-propulsion assay, indicating a favorable role in restoring gut motility. However, the estrous cycle was not monitored, which might be a limitation since ovarian hormones can affect gastrointestinal function. Estrogen and progesterone fluctuations are known to modulate GI motility, e.g., estradiol can delay gastric emptying, whereas progesterone can accelerate it [35]. By randomizing animals to groups, we aimed to evenly distribute any cycle-related effects, but some variability in outcomes could stem from this factor. This observation highlights that future investigations should address sex and hormonal cycle influences, as PPAR-γ’s effects on intestinal function might differ by sex or estrous stage.

Lactose is the main carbohydrate in human and mammalian milk, which requires enzymatic hydrolysis by lactase into D-glucose and D-galactose before it can be absorbed [9]. Previous research found that transcription factors, including Cdx-2, hepatocyte nuclear factor (HNF) family, GATA factors, and Oct-1, directly regulate LCT expression [36,37]. Recent studies on Caco-2 cells and rat model confirmed that ROSI, as the PPARγ agonist, was able to increase the expression and activity of LCT in both the proximal small bowel of rodents and human Caco-2 enterocyte-like cells [17]. Mechanistically, Fumery et al. identified the DR2 motif at −223/−210 bp as the response element in the *LCT* gene promoter with ~2-fold-increased PPARγ occupancy upon rosiglitazone stimulation. However, the direct relevance in food allergy animal models has not been examined. We found that food allergies can induce secondary lactose intolerance due to intestinal damage and mucosal erosion in rats, leading to a decline in LCT activity and expression. Ligand-activated PPAR-γ signaling can reverse this trend. Additionally, inhibiting PPAR-γ signaling in rats with GW9662 further decreased LCT expression and activity, indicating that PPAR-γ activation appears to help restore lactase expression in the gut of FA rats; this may result from a combination of direct transcriptional upregulation (especially for the *LCT* gene) and indirect effects due to alleviation of inflammation [14]. Across epithelial and animal models, ROSI has been reported to attenuate intestinal inflammation while supporting epithelial barrier integrity, including evidence from IECs [38], rat models [39], porcine models [40], and even human clinical trials [41]. Lactase is a brush-border enzyme of differentiated small-intestinal enterocytes; thus, epithelial injury and inflammation that promote villus blunting/crypt hyperplasia are expected to reduce lactase capacity, making barrier repair indirectly favorable for preserving/restoring lactase function.

SGLT1 and GLUT2 proteins facilitate the primary active transport of glucose and galactose across the intestinal epithelium [42]; mutations in the SGLT1 gene (*SLC5A1*) can lead to glucose/galactose malabsorption [43], while GLUT2 defects (Fanconi–Bickel syndrome) impair glucose and galactose handling and may lead to intolerance [44]. Our results demonstrate a significant downregulation in the expression and activity of SGLT1/GLUT2 during food allergies. Given that GLUT2 expression increases as enterocytes migrate up from the crypt to the villous tip [45], villous atrophy associated with food allergies inevitably reduces their expression, corroborating our experimental findings. The differential response of SGLT1 and GLUT2 to PPAR-γ activation was noteworthy: SGLT1 was more robustly upregulated than GLUT2. This discrepancy is likely connected to their distinct localization and functional roles. SGLT1 is a brush-border transporter expressed on the apical side of mature enterocytes, whereas GLUT2 is normally located on the basolateral membrane of enterocytes, which facilitate glucose exit to the bloodstream [46], though it can also translocate to the apical side under high luminal sugar conditions [47]. Because Rosi has been reported to alleviate intestinal injury and increase jejunal villus height, the marked reduction in SGLT1 in OVA-sensitized rats (Figure 4B, OVA vs. Ctrl) may reflect loss or dysfunction of mature villus enterocytes, with restoration of villus architecture and differentiation contributing to its rebound after rosiglitazone treatment. In contrast, GLUT2 may be less sensitive to villus-tip loss or to short-term changes in epithelial differentiation. ROSI has been reported to increase GLUT2 expression in INS-1 cells after 24 h treatment [48]. Similarly, under lipotoxicity, endoplasmic reticulum stress, and inflammatory conditions, pioglitazone upregulated GLUT2 in mouse insulinoma MIN6 β-cells [49]. Therefore, the ability of rosiglitazone to preserve GLUT2 expression under inflammatory conditions may partly explain the observed alleviation of intestinal dysfunction and lactose malabsorption in experimental food allergy models.

Considering the altered expression and activity of SGLT1, GLUT2, and LCT in our study, food allergies can reduce glucose and galactose transport; activated PPAR-γ signaling might partially reverse this while enhancing lactose absorption in the intestines. However, pharmacological activation of PPAR-γ is not devoid of side effects. Rosi has known off-target and systemic effects such as weight gain, edema/fluid retention, bone loss, and potential cardiovascular risks, which pose translational limitations [50,51]. These safety concerns motivate interest in approaches that enhance PPAR-γ activity more physiologically and with less systemic exposure. An alternative therapeutic approach could involve gut-restricted PPAR-γ modulators like 5-aminosalicylic acid (mesalamine), an established colitis treatment, which exerts its anti-inflammatory effect by activating PPAR-γ in colonic epithelial cells. This local activation avoids systemic exposure that occurs with drugs like rosiglitazone [52]. In addition, microbiota-activated PPAR-γ signaling represents a homeostatic pathway that mitigates lactose intolerance [53,54], and certain gut microbial metabolites like butyrate and propionate can act as natural ligands or stimulators of PPAR-γ in intestinal epithelial cells to improve epithelial health and barrier function [55,56]. Together, these gut-restricted strategies represent promising ways to utilize PPAR-γ as a target to alleviate lactose intolerance induced by food allergy, while potentially reducing systemic off-target effects.

## Figures and Tables

**Table 1:** List of primer sequences used in qRT-PCR.

Gene	Primer Sequences (Forward)	Primer Sequences (Reverse)
LCT	GGACATACTAGAATTCACTGCAA	GGTTGAAGCGAAGATGGGACG
GAPDH	CATGGTCTACATGTTCCAGT	GGCTAAGCAGTTGGTGGTGC

## Data Availability

The original contributions presented in this study are included in the article. Further inquiries can be directed to the corresponding author.

## Abbreviations

The following abbreviations are used in this manuscript:

FA	Food allergy
PPAR-γ	Peroxisome proliferator-activated receptor gamma
GLUT1	Glucose transporters 1
SGLT2	Sodium-dependent glucose transporters 2
LCT	Lactose
IECs	Intestinal epithelial cells
Rosi	Agonist
PBS	Phosphate-buffered saline
